# Cryopreservation of virulent *Acinetobacter baumannii* to reduce variability of *in vivo* studies

**DOI:** 10.1186/s12866-015-0580-8

**Published:** 2015-11-02

**Authors:** Travis B. Nielsen, Kevin W. Bruhn, Paul Pantapalangkoor, Justin L. Junus, Brad Spellberg

**Affiliations:** Department of Molecular Microbiology and Immunology, Keck School of Medicine at the University of Southern California (USC), Los Angeles, CA USA; Department of Medicine, Keck School of Medicine at USC, Los Angeles, CA USA

**Keywords:** *Acinetobacter baumannii*, Cryopreservation, Frozen, Inoculum, Virulence

## Abstract

**Background:**

Microbiological assays require accurate and reproducible preparation of bacterial inocula. Inocula prepared on different days by different individuals can vary significantly from experiment to experiment. This variance is particularly problematic for Gram-negative bacterial infections, for which threshold effects can result in marked variations in host outcome with minor differences in inocula.

**Results:**

We compared the accuracy of traditional methods versus using frozen stocks for preparing *Acinetobacter baumannii* inocula for infection in mice. Standard inoculum preparation resulted in substantial variability, both with respect to the actual inocula achieved compared to the targeted inocula, and with respect to the *in vivo* outcome resulting from similar inocula. Cryopreservation of the bacteria resulted in no significant decrement in growth of the bacteria. Furthermore, preparation of multiple infectious inocula from a frozen stock significantly improved the accuracy of the achieved inocula, and resulted in more reproducible *in vivo* outcomes from infection. Frozen stocks reduced inter-experiment variability associated with inoculum preparation, displayed no significant loss of growth capacity, and maintained virulence, increasing the reliability of infection.

**Conclusions:**

Frozen stocks require considerably less time to prepare and enhance reproducibility of *in vivo* experimental results when infecting with *A. baumannii*.

## Background

Microbiological studies require the frequent preparation of bacterial inocula with consistent and well-defined characteristics. Traditional methods of preparing bacterial inocula often involve growing an overnight culture, passaging a sub-culture, washing out the bacterial growth media, measuring the optical density (O.D.) at a wavelength of 600 nm and using a correlative factor to estimate the bacterial concentration, and finally plating serial dilutions on agar to measure the actual number of colony-forming units (CFUs)/mL. These methods are laborious, time-consuming, and often not completely reproducible with respect to quantity and quality of the inocula prepared. Furthermore, since colonies plated on agar cannot be counted until the following day, estimates of the bacterial number in inocula used for experiments must be based solely upon the O.D. reading of cultures that often undergo additional dilution or manipulation prior to use.

Over the course of many experiments in which we prepared bacterial inocula for injection into mice, we encountered variability from experiment to experiment that was particularly problematic given the threshold effect that Gram-negative bacteria have in virulence, in which minor changes in inocula can lead to substantial differences in host outcome. There are several likely sources of variability in traditional methods of bacterial preparation. Fresh preparations require that cultures be initiated from individual colonies from agar plates of varying ages. Spectrophotometric measurements of washed cultures introduce an inherent level of variability. Dilutions of cultures to adjust the concentration of working inocula and to plate out appropriate numbers of bacteria for CFU counting are also sources of intrinsic variance. We therefore postulated that we could eliminate many of these potential sources of variability by utilizing a single, large bacterial culture that was cryopreserved in identical aliquots, and therefore increase reproducibility between experiments [1, 2].

*Acinetobacter baumannii* is a multidrug-resistant, clinically relevant Gram-negative bacterium that causes nosocomial infections [3, 4]. To the best of our knowledge, the majority of pathogenesis studies of this organism (as well as many other Gram-negative pathogens), utilize fresh subcultures and O.D. measurements to quantitate and prepare bacterial inocula for *in vivo* challenge experiments. As a model organism, we chose a specific hyper-virulent isolate of *A. baumannii* that results in a lethal bloodstream infection in susceptible mouse strains following intravenous tail vein injection [5, 6]. We used this hyper-virulent isolate to compare different methods of bacterial inocula preparation, with regards to their effect on *in vitro* growth rates and *in vivo* induction of mortality.

## Results and discussion

**Fig. 1:**
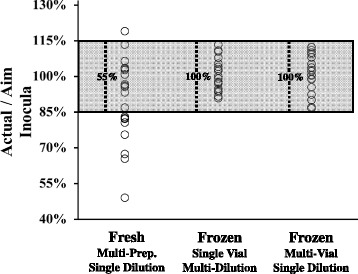
Multiple inocula preparations from fresh overnight cultures display greater variability than inocula preparations from frozen stocks. The ratio of “Actual” bacterial numbers (as measured by CFU counts one day following preparation) to “Aimed-for” concentration was used as a measure of the cumulative accuracy of each method. For fresh preparations, the “Aimed-for” concentration values of washed subcultures were estimated by determining OD_600_ readings. For frozen vials, the “Aimed-for” concentration values were calculated by using the known, predetermined concentrations of the batch. We typically aim for < +/−15 % variance from the targeted inoculum (as denoted by the shaded region). Numbers within the shaded region denote the percentages of the 20 measurements per group that fell within the 15 % range

Over the course of several weeks, different individuals prepared multiple individual inocula of *A. baumannii* HUMC1, a highly virulent strain that we have used in multiple previous publications [5, 7, 8]. We used a traditional, standard method of culturing a freshly streaked colony overnight in a suitable broth medium, subculturing it until log-phase growth, and estimating the bacterial concentration using an O.D. reading of a washed sample. Bacteria resuspended in PBS were then adjusted to a target (“aimed-for”) concentration based on the O.D. reading, and serial dilutions of these working dilutions were made to allow plating of a countable number of CFUs. “Actual” numbers of CFUs in the working dilutions were determined by counting colonies on the agar plates the following day. Multiple repeats of this protocol resulted in a wide range of “Actual”/“Aimed-for” ratios, demonstrating a high degree of variability from day to day when utilizing fresh cultures (Fig. 1).

We then cryopreserved a single large culture of *A. baumannii* in identical aliquots of freezing media at a defined bacterial concentration. Vials were thawed on various days and serially diluted to an appropriate concentration for agar plating. Multiple dilutions from the same single vial displayed a range of variability (quantified by Actual/Aimed-for ratios) that was significantly narrower than the variability arising from different, freshly prepared inocula (Fig. 1). Dilutions from multiple frozen vials displayed a similar range of values. Thus, preparing inocula from frozen batch vials allows more reproducible, accurate preparation of bacterial counts than preparing inocula from fresh cultures.

**Fig. 2:**
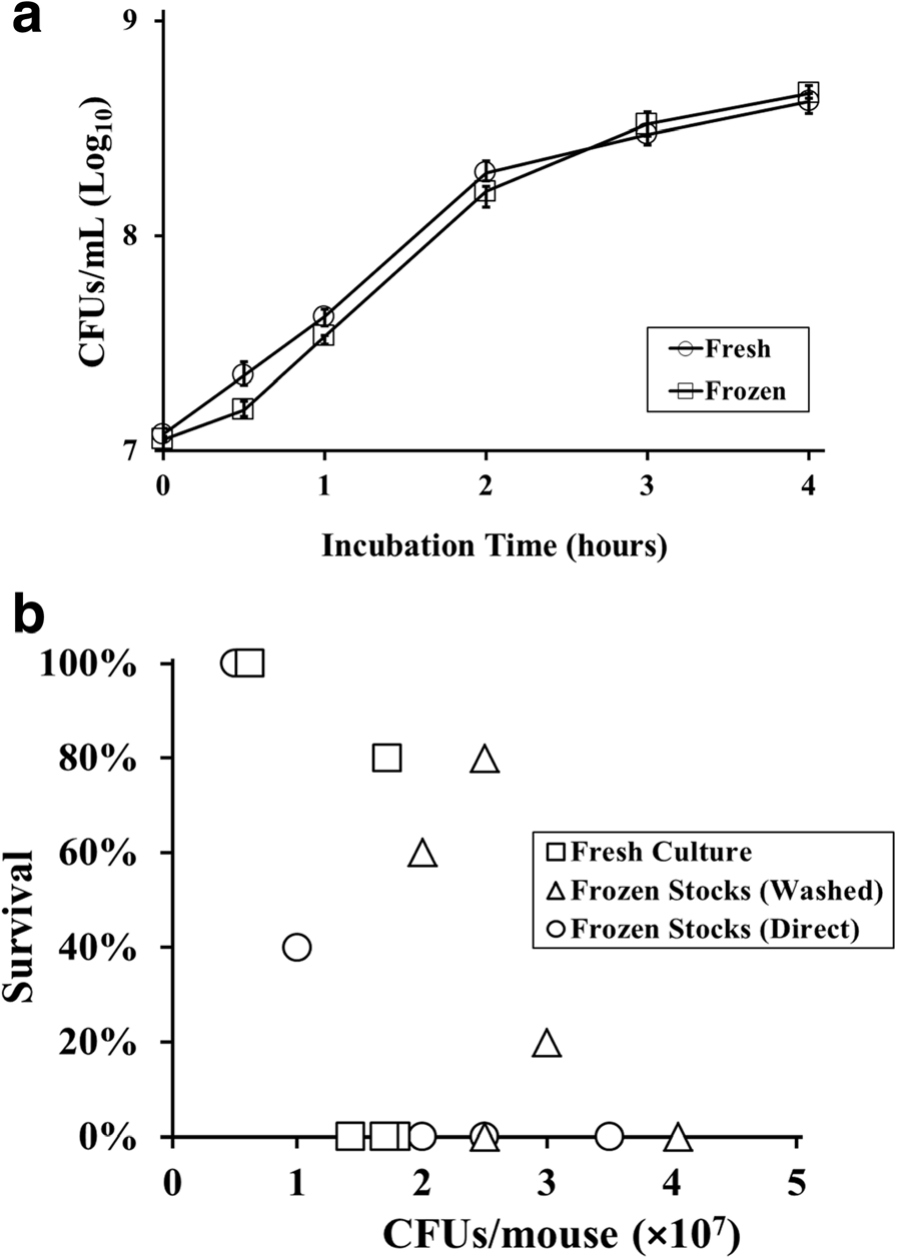
Frozen and freshly-prepared inocula display similar growth and virulence characteristics, but only frozen stocks diluted directly (not washed) gave consistent survival results. **a** *In vitro* growth curves of subcultures started from fresh overnight cultures and frozen vials are similar. TSB cultures were seeded with equal numbers of fresh or frozen bacteria, and aliquots were removed at the time points indicated. Serial dilutions were plated to measure CFUs. Results are combined from six independent experiments. Median values are plotted with error bars denoting interquartile ranges. **b** Multiple preparations of bacterial inocula were prepared on different days for intravenous injection into mice, at the doses shown on the x-axis. Freshly-prepared inocula (“Fresh Culture”, squares) were quantitated based on O.D. readings on the day of preparation. Alternatively, frozen stocks were used to prepare infectious inocula, either by washing 3x to remove trace amounts of glycerol (“Washed”, triangles), or by directly diluting the frozen stock into PBS for injection (“Direct”, circles). Frozen stock inocula were based on the known concentrations of bacteria per vial following freezing. Each symbol represents a single preparation, injected into *N* = 5 mice per group on a different day. Survival percentages are shown on the y-axis

We next asked whether cryopreservation impacts the growth characteristics of *A. baumannii in vitro*. We compared two sub-cultures of *A. baumannii* HUMC1: one started from a fresh overnight culture and the other started from a frozen stock vial of bacteria. Multiple growth curves of each subculture, performed several times under the same conditions on different days, demonstrated that, despite a slight, early lag in growth compared to the freshly inoculated culture, there were no significant differences between the curves following the 1 h time point (Fig. 2a). These results demonstrated that cryopreservation of *A. baumannii* does not result in a significant change in growth pattern *in vitro*.

To determine if *A. baumannii* frozen stocks maintained similar levels of virulence in our animal model, we prepared several inocula on different days from fresh subcultures, utilizing O.D. readings to estimate bacterial concentration prior to making necessary dilutions. We used C3H/FeJ mice because we have found that this strain is susceptible to *A. baumannii* infection while other inbred strains (e.g., BALB/c, C57BL/6, etc.) are highly resistant and require much higher infectious inocula to cause lethality [5, 7, 8]. Groups of 5 mice each were intravenously injected with these inocula to determine an approximate LD_100_ (Fig. 2b, squares). Although three separate inocula, calculated to be 1.4 × 10^7^, 1.7 × 10^7^, and 1.8 × 10^7^, all resulted in 0 % survival, another inocula calculated to be 1.7 × 10^7^ resulted in 80 % survival, highlighting the difficulties of reproducing *in vivo* results by preparing inocula on different days.

We compared these results to those generated after preparing inocula from frozen vials on different days. Initially we pelleted and washed each vial’s contents to completely remove freezing media, then resuspended the pellet in an appropriate concentration of PBS for injection, using the CFU/mL concentration predetermined by plating of multiple vials. Five independent inocula prepared by this method were injected into mice at different times. As with freshly prepared inocula, doses with very similar calculated CFUs/mL had different survival levels (80 % vs. 0 % for separate doses of 2.5 × 10^7^/mouse) (Fig. 2b, triangles). These results highlight the difficulties of reproducing experiments when using inocula that require multiple pelleting and washing steps to prepare.

In an attempt to further reduce variability, we eliminated the pelleting and washing steps and directly diluted the thawed frozen vials to various final concentrations in PBS for injection. Injections of directly diluted frozen vials into mice (Fig. 2b, circles) resulted in LD_100_ calculations that were similar to inocula prepared from both washed frozen stocks, and freshly prepared cultures. Thus the small amount of glycerol in directly diluted stocks were not toxic and did not impact the virulence of *A. baumannii* in our model, which is consistent with the known safety profile of glycerol [9]. Furthermore, inocula that were calculated as equal to or greater than 2.0x10^7^/mouse uniformly resulted in 0 % survival, while less than 2.0x10^7^/mouse resulted in higher survival percentages (Fig. 2b, circles). Thus, by utilizing frozen stocks, and eliminating pelleting and washing steps that could lead to variability, we observed more reproducible survival results in experiments performed on different days. Importantly, we have tested frozen vials that were prepared and frozen more than 18 months prior to use, and found similar levels of both CFUs/mL and lethal inocula as found with earlier preparations, suggesting that viability of frozen stocks does not detectably decrease over this period of time.

## Conclusion

The preparation of a single, large stock of bacterial culture that can be frozen in identical aliquots and used repeatedly in different experiments offers many theoretical and practical advantages over the more time-consuming method of preparing inocula each time from freshly grown cultures. For *in vivo* studies of infection caused by Gram-negative bacilli, accuracy in achieving the targeted infectious inocula can be critical, because of the threshold effect such organisms have in triggering death from sepsis. We have found that this threshold phenomenon is particularly true for *A. baumannii.* A 50 % drop off in inocula changes an LD_100_ into a non-lethal dose.

Here we show that *A. baumannii* frozen aliquots have similar growth characteristics to fresh subcultures, as well as similar virulence levels, as measured by lethal thresholds in a mouse model of systemic infection. Inocula prepared from frozen aliquots require significantly less time to prepare, and can be directly diluted to give known bacterial concentrations, without the need for centrifuging and washing bacterial pellets. Most importantly, they enable a more accurate achievement of targeted bacterial inocula, and thus enable reproducible *in vivo* post-infection survival rates, without reliance on spectrophotometer OD_600_-based estimations, serial dilutions, or manual counting of colony-forming units on agar plates. Replication of experimental results becomes more attainable through the use of identical sub-cultures and eliminates a major source of variability.

## Methods

### Preparation of inocula from fresh cultures

A single colony of *A. baumannii* HUMC1, a carbapenem-resistant clinical blood and lung isolate from an in-patient with bacteremic ventilator-associated pneumonia, at Harbor-UCLA Medical Center in 2009 [6], was used to seed an overnight culture in tryptic soy broth (TSB). Bacteria were passaged to log-phase at 37° for 3 h following a 1:100 subculture in 10 mL TSB, pelleted, and resuspended in 10 mL of fresh PBS. Bacterial density was estimated by measuring the OD_600_ of various dilutions (to adjust to within a linear range of the spectrophotometer), and a relationship of [OD_600_ = 4 × 10^8^ CFU/mL] was used to calculate an approximate bacterial concentration of the resuspended preparation. Based on this estimate, serial dilutions (10-fold) were made and appropriate dilutions were plated and spread on agar plates, to allow counting of CFUs the following day, and determination of the actual bacterial density.

### Cryopreservation of *A. baumannii* and preparation of inocula from frozen stocks

An overnight culture of HUMC1 was grown as above, subcultured to log-phase in 1 L of TSB, pelleted, and resuspended at an approximate concentration of 1 × 10^10^ CFUs/mL (based on OD_600_ readings). Following mixing to ensure even homogenization of the culture, 0.6 mL aliquots of the bacterial suspension were placed into pre-labeled 1.5 mL cryotubes containing 0.6 mL of a sterilized 50 % glycerol. Vials were evenly mixed and immediately stored in a −80 °C freezer. To determine the exact bacterial concentration of these frozen stocks, multiple vials were thawed, serial dilutions were plated to quantitate CFUs/mL, and an average value was obtained.

### Growth curves

An overnight culture of a freshly streaked colony of HUMC1 was prepared as above and the concentration was estimated by OD_600_ reading. Simultaneously, a frozen stock vial of known concentration was thawed. Each was diluted to 2.5 × 10^9^ CFUs in 10 mL PBS, then added to 240 mL TSB. These cultures were incubated at 37 °C with 200 rpm shaking, and 1 mL aliquots were sampled at time points 0, 0.5, 1, 2, 3, and 4 h. CFUs were determined by plating serial dilutions on TSA plates.

### *In vivo* virulence testing

Male C3HeB/FeJ mice (Jackson Labs) were used at 10–12 weeks of age. This strain of mouse was chosen based on its well-described susceptibility to lethal infection with *A. baumannii* [5, 7, 8]. Mice were injected intravenously via the tail vein with various inocula of bacteria resuspended in 250 μL PBS. Mice were monitored for signs of infection and euthanized if moribund. All animal experiments were approved by the Institutional Committee on the Use and Care of Animals at the Keck School of Medicine at USC, following the National Institutes of Health guidelines for animal housing and care.

## Abbreviations

CFU: Colony Forming Units
LD_100_: Lethal Dose for 100 %
O.D.: Optical Density
PBS: Phosphate-buffered Saline
TSA: Tryptic Soy Agar
TSB: Tryptic Soy Broth
